# Potential biomarkers of chronic low-dose radiation exposure for nuclear medicine technologists

**DOI:** 10.1080/09553002.2025.2470225

**Published:** 2025-03-18

**Authors:** Irene Mbutu-Austin, Bijan Modarai, Elizabeth Ainsbury, Samantha Y. A. Terry

**Affiliations:** a School of Biomedical Engineering and Imaging Sciences, King’s College, London, UK; b NIHR Health Protection Research Unit in Chemical and Radiation Threats and Hazards, King’s College London, London, UK; c Academic Department of Vascular Surgery, School of Cardiovascular and Metabolic Medicine and Sciences, King’s College, London, UK; d Radiation, Chemical and Environmental Hazards Directorate, UK Health Security Agency, Oxford, UK

**Keywords:** Nuclear medicine, biomarkers, ionizing radiation, biomonitoring, low dose exposure, genotoxic

## Abstract

**Purpose:**

Nuclear medicine is the fastest growing segment in imaging due to an increase in demand for procedures, development of advanced scanners and new radioactive tracers. Technologists are exposed to radiation throughout the workday. Key protection approaches are time, distance, and shielding; these can be difficult to achieve since patients are usually the main source of radiation and close contact is required. Technologists in general nuclear medicine receive annual effective doses of approximately 0.1 mSv. Doses in positron emission tomography (PET) imaging can be close to 6 mSv. Without appropriate radiation protection measures, finger doses from handling PET radiopharmaceuticals can exceed the annual dose limit of 500 mSv. Estimates of health risks from low dose-rate exposures are extrapolated from risk coefficients calculated from Japanese atomic bomb survivors. Effects of chronic exposure are obtained from nuclear workers and radiotherapy patients. This review aims to consolidate existing research in biomarkers of low dose radiation exposure to determine whether they may form a part in occupational health monitoring.

**Conclusions:**

The link between chronic low-dose exposure in nuclear medicine technologists and health risks using radiation-related biomarkers as a proxy remains relatively unexplored. Further work is needed to identify and characterize biomarkers in technologists.

## 1. Introduction

### 1.1. Nuclear medicine and PET imaging technologists

Medical imaging makes use of ionizing radiation. In radiology, an external source produces X-rays, which interact with tissues in the patient’s body creating an image. In nuclear medicine, radioactive tracers are administered to the patient, either through ingestion, inhalation or intravenous injection and the emitting gamma rays are then used to create images. Positron emission tomography (PET) is a gamma imaging technique from positron-emitters. Gamma rays produced from the annihilation reaction of a positron and an electron have high energies of 511 keV and have a penetrating ability greater than that of the 140 keV emissions from ^99m^Tc-based compounds used in general nuclear medicine (Roberts et al. 2005).

The occupational exposure to radiation for technologists occurs when handling and administering the radioactive tracers to the patients, with further radiation exposure from the patients after they have received the tracer (Zakeri and Hirobe 2010). With PET imaging, there are also added radiation safety concerns because of the higher radiation and longer patient uptake times (Zeff and Yester 2005). Technologists working in general nuclear medicine typically receive annual effective doses of 0.1 mSv, but doses for those working in positron emission tomography (PET) imaging are frequently close to 6 mSv. If appropriate radiation protection measures are not in place, finger doses from drawing up and administering PET radioactive tracers can exceed the annual dose limit of 500 mSv (ICRP 2019). In addition to the general body exposure received from patients, the use of unsealed sources presents high exposure to the hands as the technologists prepare, draw up, and inject the radiopharmaceuticals (Carnicer et al. 2011).

### 1.2. Biological effects of ionizing radiation

The biological effects of ionizing radiation result from the ionization of proteins or DNA within the body, which may alter or destroy the functions of proteins, cells, or tissues. Health effects from radiation exposure can be classified for radiation protection purposes as either tissue reactions or stochastic effects (Domenech 2017). Tissue reactions, also known as deterministic effects, have a threshold radiation dose after which the response of the cells is dose related with the frequency of cell injury and severity of damage increasing with radiation dose (Elgazzar and Kazem 2015; Domenech 2017). Stochastic effects usually occur following exposure to low-dose radiation, sometimes over several years, and are generally considered carcinogenic and can lead to genetic defects in offspring (Florida Uo 2012; Elgazzar and Kazem 2015). Stochastic effects may only be observable some years after exposure. The effects of radiation exposure associated with nuclear medicine occupational exposures are not well studied to date, however there are some studies (Gharibdousty et al. 2017; Miszczyk et al. 2019) that show an increase in chromosomal aberrations in blood lymphocytes of nuclear medicine technologists compared to subjects not exposed to radiation. These studies are reviewed in more detail below.

### 1.3. Radiation dose limits

To ensure that no one is exposed to ionizing radiation at harmful deterministic or stochastic levels, the International Commission on Radiological Protection (ICRP) recommends occupational and public exposure dose limits alongside guidance in radiation protection. In the UK, the Ionizing Radiation Regulations 2017 (IRR17) implement the ICRP recommendations and recommended dose limits. In 2012, the ICRP reduced the dose limits to the eyes from 150 to 20 mSv per year (Boal and Pinak 2015) to reduce the risk of cataract formation in radiation workers. The previous dose limit had been based on single acute radiation exposures and earlier epidemiological studies which may not have had sufficient follow-up (Clement et al. 2012). Radiation dose limits are therefore still able to be altered based on scientific evidence.

### 1.4. Radiation safety measures in nuclear medicine

Radiation safety measures in nuclear medicine are based on the principle of keeping radiation dose to people as low as reasonably practicable (ALARP in the UK; ALARA elsewhere). UK nuclear medicine technologists work with the ALARP principle, by employing the time, distance and shielding concept to reduce radiation dose (Bolus 2008). Reducing the amount of time spent around radiation sources will reduce the amount of radiation exposure. The inverse square law is also applied to reduce radiation exposure as the exposure is inversely proportional to the square of the distance from the source of radiation (Bolus 2008). Examples of increasing distance to the radiation source can include handling radioactivity using tongs. Shielding is achieved by storing radioactive vials in lead pots, drawing up behind lead or Perspex shields and using syringe shields when injecting. The use of syringe shields when drawing up and injecting patients reduces exposure to the hands significantly, but dose limits could still be exceeded if radiation protection measures are not sufficient (Carnicer et al. 2011).

Shielding the body is more difficult because frequent wearing of lead aprons which are heavy will affect the technologists’ mobility and may result in back problems (Pelz 2000; Aminian et al. 2017). Shielding using lead aprons is also not effective for positron-emitters like those used for PET imaging. Lead shielding can also lead to more radiation exposure to personnel because of the annihilation effects of the positrons with the lead (Deb et al. 2015). Protection of technologists is complicated by the fact that 90% of the technologists’ occupational dose comes from close interaction with the patients (Zeff and Yester 2005). Patient interaction is difficult to avoid, and more time needs to be spent with patients who are particularly unwell. Even when applying all necessary precautions as described above, depending on a clinic’s workload and department radiation protection standards, maximum doses to the hands may still surpass dose limits, especially for technologists working with PET isotopes (Carnicer et al. 2011).

Regulation 21 of IRR17 requires employers to designate an employee as classified based on their potential exposure to ionizing radiation because of the work the individual is required to undertake. Nuclear medicine technologists have, over the last five years, become designated classified radiation workers in the UK, because the potential dose accrued from an unnoticed contamination of a glove by a droplet or spill of a radioactive tracer combined with normal working practices could exceed the occupational exposures dose limits.

The demand for nuclear medicine procedures is increasing up to 5% annually (WNA 2022). In the UK, there has been a 12.6% increase in PET/CT procedures that use higher energy emitters (Irwin et al. 2022). With improvements in PET technology, new-generation PET cameras (e.g. total body PET scanners) have also now been developed, increasing throughput and potentially increasing technologist doses because of this higher workload (Roberts et al. 2005). This increase in demand for nuclear medicine and especially PET procedures will increase the dose exposure to nuclear medicine technologists. It is therefore paramount that we understand the long-term biological effects of radiation in technologists.

Here, a literature search was conducted for long-term longitudinal studies on medical radiation workers as well as for studies carried out between 2000 and 2021 that employed biological methods for dose assessment. Only studies in English were included.

## 2. Health risks from occupational ionizing radiation

The possibility of long-term effects on people receiving low chronic exposure is of public health significance. It is usually necessary to observe irradiated populations for years and employ biostatistical and epidemiologic methodology. This is complicated by the latent period where a radiation-induced increase of a disease may not be noticed or recorded unless the study is continued for long periods of time, usually many years (Florida Uo 2012). The current estimates of health risks from low dose-rate exposures are mostly extrapolated from risk coefficients calculated from Japanese atomic bomb survivors (Yoshinaga et al. 2004; Linet et al. 2010). The effects of chronic radiation exposures have been obtained from nuclear workers and from studies of radiotherapy patients but exposures are generally at high dose rates (Linet et al. 2010). There are no long-term epidemiological follow-up studies assessing cancer risks in medical radiation workers (Yoshinaga et al. 2004; Linet et al. 2010) despite there being an increase in use of radiation in modern medical diagnostic settings. There are very few studies assessing risks to nuclear medicine technologists because of the scarcity of clinical, epidemiologic, and dosimetry studies and the complexity of dose estimation from internal and external radiation exposures (Linet et al. 2010).

Studies described below are amongst the most relevant to technologists working in nuclear medicine and PET imaging with the caveat that studies with only general radiologists and radiologic technologists may not be generalizable to nuclear medicine technologists as technologists receive higher cumulative radiation exposure from the wider range of photon energies of the radioisotopes (Linet et al. 2010; Kitahara et al. 2015).

### 2.1. Ongoing study in USA

There is currently an on-going longitudinal study in the USA, which follows radiological technologists employed between 1926-1982. The study is investigating potential radiation-related health effects from low-dose occupational radiation exposure. Four surveys have been sent out to medical imaging workers so far. Review of the first three surveys from 1983-1989, 1994-1998, and 2003-2005, found that technologists who performed nuclear medicine procedures had modestly elevated risks for some health outcomes compared to those that did not perform nuclear medicine procedures. These included female breast cancer squamous skin cell carcinoma, and myocardial infarction) (Kitahara et al. 2015). The researchers in this study suggested that these modest health risks require longer follow-up and should also include individual radiation dose estimates of the technologists with more details on the types of procedures performed and radioactive tracers used.

A fourth survey was carried out between 2012 and 2014, which now also included nuclear medicine technologists. The results released so far show that annual doses to USA radiologic technologists who work in general radiology declined during a 36-year period from an average of 0.6 mSv to below the limits of detection but doses for nuclear medicine remained high at between 1.2 mSv and 2.2 mSv for those working in PET imaging (Villoing et al. 2021). In terms of health effects, an increased risk of cataracts was observed among technologists who had performed a nuclear medicine procedure at least once. 7,137 incident cataracts were reported from 42,545 technologists. A significantly increased risk of cataract (Hazard ratio; HR, 1.08; 95% confidence interval [CI]: 1.03, 1.14) was observed in technologists who performed a nuclear medicine procedure at least once as opposed those who had not. Specific risks of cataracts for those who had performed a diagnostic or therapeutic nuclear medicine procedure had a HR of 1.07 (95% (CI: 1.01, 1.12) and 1.10 (95% CI: 1.04, 1.17), respectively (Bernier, Doody, et al. 2018; Bernier, Journy, et al. 2018). This survey observed no increased risk of cancer incidence or cancer deaths in technologists who performed nuclear medicine procedures. The authors suggest that a longer follow up with larger sample size may be necessary for more precise estimation or elimination of cancer risk (Bernier, Doody, et al. 2018). The data relating to nuclear medicine technologists unfortunately does not include nuclear medicine technologist registered after 1982; considering the increasing demands for nuclear medicine studies, especially PET/CT, since then it would be useful to follow those certified later also to get a more up-to-date representation of nuclear medicine technologists today.

### 2.2. Japanese technologists 1969-1993

This was a retrospective cohort study that followed male technologists licensed from 1968 and born between 1897 to 1950 (Yoshinaga et al. 1999). The cohort was followed from 1969 until 1993. Key findings include an apparent high risk of hematologic cancers which suggested a carcinogenic effect of occupational radiation exposure in the long term. Extending the observation period would improve the statistical power because the background mortality of cancer is still increasing in this study population (Yoshinaga et al. 1999).

### 2.3. Danish radiotherapy workers 1968-1985

This was a longitudinal study which followed a mix of professionals who worked between 1954 and 1982 in two radiotherapy departments in Denmark (Andersson et al. 1991). The follow-up was from 1968 to 1985. The study observed no relationship between the radiation dose or years of radiation exposure and cancer risk. Skin cancer incidence increased with increasing radiation dose measured although the trend was not significant (Yoshinaga et al. 2004). This study unfortunately did not include a longer follow-up time. The measured doses were typical for these radiotherapy workers, with increased doses arising mostly when entering the treatment room before treatment is complete, or leaving the door to the treatment room ajar (Tofani et al. 1999). A lack of a dose-effect relationship in these workers might also be due to the use of the general public as the ‘control group’. This approach could lead to the healthy worker effect, where workers are generally healthier than the general public (Yoshinaga et al. 2004). This may be more pronounced in radiology technologists who may have a healthier lifestyle and have better access to medical care.

### 2.4. Chinese X-ray workers 1950-1995

This longitudinal study followed radiologists and radiographers who were employed in diagnostic X-ray from 1950 to 1980 (Wang et al. 2002) and followed through to 1995. Key findings include significantly elevated risks observed for leukemia, skin cancers, female breast, lung, liver, bladder, and esophagus cancers. The incidence of leukemia in those employed before 1970 was significantly higher than expected and cancer risk overall was higher for those employed before 1970 also. The patterns of risk correlated with number of years working with X-rays, and with age and year of initial employment. This study suggested that the excesses health risks were related to occupational exposure to X-rays.

### 2.5. Canadian radiation workers

This longitudinal study followed individuals registered in the national registry from 1950 to 1983 and followed them up to 1987 (Zielinski et al. 2009). In this study, thyroid cancer incidence was significantly elevated. It also showed that, compared to other medical workers, nuclear medicine technologists had the highest mean yearly radiation dose over the follow-up period. The study had a short follow-up time especially in those registered in the 1980s. The comparison population was again the general public.

### 2.6. US army technologists 1946-1947

This longitudinal study followed men trained as X-ray technologists during the Second World War (Jablon and Miller 1978). The follow-up was from 1946-1974. There was no evidence of an increase in incidences of cancer. Follow-up ended in the mid-1970s before the majority of the technologists were at the ages at which cancer risks are the highest which does not allow for the substantial latency period for cancer development (Linet et al. 2010).

### 2.7. US radiologists 1920-1969

This longitudinal study followed radiologists in the US registered between 1920 and 1969 (Seltser and Sartwell 1965). The follow-up was for fifty years. Elevated mortality ratio for all cancers was observed with particular elevated mortality and incidences of leukemia and lymphoma observed in the 1920–1939 cohort (Yoshinaga et al. 2004).

### 2.8. UK radiologists 1897-1997

This was a retrospective study following radiologists registered in the United Kingdom between 1897 and 1954 (Court Brown and Doll 1958) and expanded later to include radiologists registered between 1955 and 1979. The radiologists were followed up until 1997. The study showed a significant excess of cancer deaths, mainly skin cancers, pancreas and possibly leukemia in radiologists who started their careers in radiology before 1921, the year in which the first radiation protection advisory committee was formed (Court Brown and Doll 1958). There was evidence that there was a 41% excess risk of cancer mortality for radiologists who were registered for more than 40 years, possibly a long-term effect of radiation exposure in those who first registered during 1921-1954 (Berrington et al. 2001; Linet et al. 2010). There was no increased risk in those who registered after 1954, when exposures were lower (Berrington et al. 2001).

These studies, although showing elevated risks for some cancers lack, long term follow up and highlight the need for newer epidemiological studies specific to medical radiation workers including nuclear medicine technologists, especially in light of the increase in demand for procedures involving radiation. What is also clear from the studies described above is that only few studies were specific to nuclear medicine and none of these were carried out in the UK recently. Also, there is still no up-to-data and clear-cut dose-effect relationship that is relevant to nuclear medicine and PET imaging technologists. Whereas usually this relies on longitudinal follow-up studies by which time the outcomes will be too late or even out-of-date, studies incorporating biomarkers of relatively low-dose, chronic exposure in this population might be more effective at informing health risks. Standard epidemiological studies are costly but also limited at lower doses. Adding biomarkers of individual exposure to epidemiological studies would improve the studies to create molecular epidemiological studies which would expand our understanding of low doses and low dose rate effects (Hall et al. 2017) There is also a point to be made that perhaps any such biomarkers might also make a good monitoring tool for exposure, alongside dosimetry values determined by live and/or passive dosimeters, that could provide more personalized risk measurements to the individual. This would also minimize reliance on dosimetry values that perhaps underestimate dose received, due to improper wearing (Sari-Minodier et al. 2007; Gharibdousty et al. 2017) or doses received being under the detection limit.

## 3. Biomarkers of radiation exposure

### 3.1. Studies investigating early biomarkers of radiation exposure

There have been several studies relating DNA- and chromosome-based biological markers to radiation exposure; Table 1 summarizes work carried out in nuclear medicine technologists, whereas Table 2 includes studies in which nuclear medicine technologists are mentioned as a separate participant group.

**Table 1.** Studies around biological effects from radiation in nuclear medicine workers.

Study	Country	Results	Limitations and main conclusions
(Miszczyk et al. 2019)	Poland	Micronuclei *Nuclear medicine* 24.90 ± 11.56 *Controls* 19.19 ± 6.14 (*p <* .05)	Main conclusion: The increased chromosome fragments and micronuclei frequencies in nuclear medicine technologists may suggest potential future genetic risks and indicate a need for more studies with a larger number of technologists. Limitations: Technologists used Iodine only, which has high energy emissions, more so than the rest of nuclear medicine.
(Gharibdousty et al. 2017)	Iran	Micronuclei *Nuclear medicine* 25.82 ± 8.67 *Controls* 10.52 ± 6.83 (*p <* .0005)	Main conclusion: The frequencies of micronuclei were significantly higher in the workers than in the control. This might be due to an accumulation of initial DNA damage in workers exposed to chronic radiation. Cytogenetic monitoring is a valuable addition to film dosimetry after low dose radiation exposure and for more accurate occupational risk assessment. Limitations: Male participants only.
(Djokovic-Davidovic et al. 2016)	Serbia	Chromosome aberrations (CA) compared to the previous checkup 74.4 % no increased frequency of CA. 15.6 % showed increased frequency. 61% increase in acentric fragments.	Main conclusion: Long-term exposure to low doses of ionizing radiation may cause carcinogenesis. Monitoring the frequency of chromosomal aberrations (with micronucleus test), hematological tests and the received dose of ionizing radiation are necessary factors for occupational health evaluation. Limitations: No control group. No exact dose reading given.
(Vral et al. 2016)	Belgium	Micronuclei per 1000 cells *Nuclear medicine* 15.41 *Controls* 15.28 (*p =* .85)	Main conclusion: Chronic radiation exposure may have genotoxic effects. Cytogenetic monitoring in addition to physical film dosimetry would be useful as a routine biomonitoring method for medical workers who receive high occupational radiation doses. Limitations: No standard deviations given for micronuclei frequency.
(Terzic et al. 2015)	Serbia	Micronucleus *Nuclear medicine* 18.39 ± 10.81 *Controls* 5.61 ± 3.92 (*p =* .001)	Main conclusion: Chronic low dose ionizing radiation exposure causes increased micronuclei frequency, despite absorbed doses being below the regulatory limits. The most important predictors of the micronuclei formation and frequency in nuclear medicine technologists are the length of years working as a technologist and the received annual doses. Limitations: Control numbers of participants less than technologist numbers.
(Dobrzyńska et al. 2014)	Poland	Comet Tail moment *Nuclear medicine* 1.28 ± 1.44 *Controls* 0.30 ± 0.44 (*p =* .013) % DNA *Nuclear medicine* 2.41 ± 1.89 *Controls* 0.78 ± 0.54 (*p =* .001)	Main conclusion: Enhanced damage to DNA in leukocytes of nuclear medicine employees still occurs despite the use of radiation protection devices. The amount of DNA damage is dependent on the kind of work carried out and although most of the DNA damage in the study is repaired, improvement of radiation protection measures should be considered. Limitations: Comet assay looks at single strand breaks which can be caused by a lot of factors so not sensitive only for radiation. No long-term damage marker, such as chromosomal damage.
(Pinto and Amaral 2014)	Brazil	Dicentrics 2 technologists with dicentrics.	Main conclusion: The true dose to the technologists will not be reflected in the dose acquired from physical dosimeters which only give dose readings about their own absorbed dose. Dose readings from physical dosimeters data may be misleading if the badge was positioned incorrectly or misplaced and left within a field of high radiation. Biological dosimetry can resolve cases of questionable dosimeter readings. Limitations: Use of odds ratio to estimate dose not the most accurate method.
(Pajic et al. 2011)	Serbia	Micronucleus *Person 1* 40 per 1000 cells. *Person 2* 19 per 1000 cells.	Main conclusion: Micronuclei in this study appear as a biological response to radiation exposure. Good radiation protection practices when handling unsealed sources of ionizing radiation, continuous education of technologists in radiation protection and regular medical and cytogenetic monitoring, will contribute to a safer work environment. Limitations: Only 2 technologists with high dosimeter readings were selected for cytogenetic analysis. No micronucleus assay in other technologists for comparison, they may have been exposed as well even with normal dosimeter readings.
(Sahin et al. 2009)	Turkey	During work Micronucleus 21.90 ± 1.71 *After 1 month vacation* Micronucleus 14.13 ± 1.25 (*p <* .001)	Main conclusion: Micronucleus frequencies were significantly higher in technologists during their working days than after their one month vacation period. There was sufficient reduction in damage after the vacation, perhaps by DNA repair or by removal of damaged cells from the blood through apoptosis. Limitations: Micronuclei formation is not specific to radiation because various physical and chemical agents, and some chronic illnesses increase the frequency of micronuclei.
(Georgieva et al. 2008)	Bulgaria	Comet assay *Control* 1.24 ± 0.09 *Technologists* <20 mSv/yr (1.41 ± 0.28) >20 mSv/yr (1.55 ± 0.38*) Induced repair after ex vivo 2 Gy exposure *Control* 0.70 ± 0.07 *Technologists* <20 mSv/yr (0.63 ± 0.17) >20 mSv/yr (0.58 ± 0.12*) *р* < 0.05 compared to control	Main conclusion: Low dose exposures had favorable effect on DNA damage and repair. DNA damage which is quickly repaired may stimulate repair systems, lead cells to be resistant to the effects of radiation and decrease oxidative stress levels. Limitations: Low numbers for control groups. Control from administration staff within same department who could potentially have contact with radioactive patients. No long-term damage marker, such as chromosomal damage.
(Kopjar and Garaj-Vrhovac 2005)	Croatia	Comet tail length *Nuclear medicine* 21.44 ± 0.14 micron *Tail length controls* 13.96 ± 0.02 micron (*p <* .01) Chromosomal aberrations mean *Nuclear medicine* 2.37 ± 0.16 *Contros.* 0.85 ± 0.09 (*P <* .01)	Main conclusion: There was a significant increase in DNA damage and increased frequencies of chromosomal aberrations in technologists compared to controls. Biological markers give information on the actual risk of radiation to exposed workers. Biomarkers also measure individual radiation damage and this would give information on individual radiosensitivity which varies between different people. Limitations: Comet assay is not specific to ionizing radiation. The exposed group was too diverse, including cleaners and engineers. It would be better to be specific to technologists.
(Joseph et al. 2004)	India	Micronucleus *Nuclear medicine* 9.80 +/- 6.20 *Controls* 7.00 +/- 3.80 (*p =* .037)	Main conclusion: The increase in micronuclei frequency between nuclear medicine technologists and controls was not significant after elimination of unhealthy individuals. This implies that the initial increase may have been due to factors other than occupational radiation exposure. The doses received by the technologists were too low for detection or alternatively any damage caused was repaired. Limitations: The control group was 27% of the sample. It would have been better to have a control group of the same sample size as the nuclear medicine sample.
(Bozkurt et al. 2003)	Turkey	Sister chromatid exchange *Nuclear medicine* 7.85 ± 1.21 *Controls* 5.24 ± 1.60 (*p <* .001)	Main conclusion: The increase in SCE frequency indicates the possibility of genotoxic effects of occupational radiation exposure. Nuclear medicine technologists should abide by good radiation protection practices and procedures. Limitations: Only looked at physicians. Technologists have more patient contact, would have been good to include technologists.
(Mozdarani et al. 2002)	Iran	Chromosomal aberrations % aberrant cells *Nuclear medicine* 6.2% − 22.18% *Controls* 1.13%	Main conclusion: Low doses of radiation exposure might not lead to DNA double strand breaks but might cause single strand breaks or base damage, which can be observed as chromatid type aberrations. Cytogenetic biomonitoring is a more sensitive and reliable method which in this study showed an increased frequency of chromatid aberrations where the film badges did not record any dose. Limitations: All male cohort with control numbers less than exposed.

**Table 2.** Studies on biological effects from radiation in general radiology workers including nuclear medicine technologists.

Study	Country	Results	Main conclusions and limitations
(Gao et al. 2020)	China	Micronucleus frequency *Radiology workers* 3% *Controls* 2% (*p* = .001)	Main conclusion: The increase in micronuclei is related to radiation dose and depends on the level of radiation exposure. Oxidative stress may be one of the mechanisms of chromosomal damage in peripheral blood cells. Limitations: Unequal numbers of controls to radiology workers. Would have been useful to divide exposed based on different modalities. Results are not easy to interpret because they are given as percentages as opposed to mean no of micronuclei with no values for uncertainty given.
(Gaetani et al. 2018)	Italy	Comet assay Accumulation of DNA damage in radiology workers. High level of repair of induced DNA damage in radiology workers.	Main conclusion: Occupational low dose ionizing radiation exposure induces DNA damage response in radiology workers. Periodic biomonitoring in addition to exposure monitoring may be advisable especially for those with a family history of cancer. Limitations: Results not easy to interpret, displayed as Tukey box plots with no data written out. Would have been useful to divide the radiology group based on different imaging modalities. No long-term damage marker, such as chromosomal damage.
(Bouraoui et al. 2013)	Tunisia	Micronucleus *All workers* 13.6 ± 4.9 *Nuclear medicine workers* 14.25 ± 7.93 *Controls* 6.5 ± 4.2 *p <* .001	Main conclusion: Despite the low levels of radiation exposure, formation of micronucleus is more frequent in technologists exposed to ionizing radiation than in the controls. Health monitoring may aid in detection of early genotoxic effects which may allow for the adoption of workplace measures such as reducing the number of hours of occupational exposure. Limitations: Lack of data on individual dosimeter because as the authors point out, most of the workers in the study did not use dosimeters.
(El-Fayoumi et al. 2012)	Saudi Arabia	Comet assay *Radiology workers* 7.44 ± 2.35 *Controls* 3.01 ± 1.33 Chromosomal aberrations *Radiology workers* 3.14 ± 0.45 *Controls* 1.10 ± 0.42 (*p <* .01)	Main conclusion: The radiology workers, despite the relatively low doses recorded, had significantly increased levels of primary DNA damage compared to control. This highlights a need for cytogenetic analysis in addition to physical dosimetry for radiation monitoring. Limitations: Lack of individual dose readings, accumulated doses do not specify period worked. Not suitable to use students in X-ray control room as control subjects since there is no guarantee they would not get some exposure.
(Ropolo et al. 2012)	Italy	Micronucleus Bi-nucleated cells *Radiology workers* 3.87 ± 2.14 Controls 3.66 ± 1.68 *Mono-nucleated cells* 1.01 vs 0.40 per 1000 cells *p* < .001	Conclusions: An increased frequency of micronuclei was observed with an increase in the accumulated radiation dose (but not affected by the length of employment). There is a potential usefulness of the micronucleus assay as a biological marker in medical surveillance. Limitations: Authors cannot attribute a meaning to the added mono-nucleated cells. No individual dose readings.
(Martinez et al. 2010)	Mexico	Comet tail length *Nuclear medicine* 92.5 ± 19.02 micron *Controls* 15.2 ± 1.92 DNA migration *Control before work. Nuclear medicine before work.* 15.5 ± 2.4 μm 54.05 ± 3.7 *Control after work. Nuclear medicine after work.* 15.2 ± 1.92 μm 92.5 ± 19.02 *p* = 0.85	Conclusions: Nuclear medicine and radiotherapy technologists had higher monthly dose exposures and more DNA damage than general radiology technologists. Longer comet tail lengths were observed in nuclear medicine implying a different type of exposure and radiation. Most of the DNA damage detected by the comet assay is repaired but some of the damage could result in stable chromosomal aberrations that may represent a long-term health risk. Limitations: Comet assay looks at single strand breaks which can be caused by a lot of factors so not sensitive only for radiation. No long-term damage marker, such as chromosomal damage.
(Zakeri and Hirobe 2010)	Iran Japan	Chromosomal aberrations *Nuclear medicine* 2.87 ± 1.4 *Controls* 1.28 ± 0.5 (*p* < 0.001) Micronucleus *Nuclear medicine* 19.7 ± 3.8 *Controls* 11.8 ± 6.5 (*p* < 0.01)	Main conclusion: The increased chromosomal aberrations and micronuclei frequencies in technologists may be a sign of potential genetic effects that may contribute to future health effects like radiation induced cancers. Biomonitoring and accurate estimation of absorbed doses in technologists exposed to occupational ionizing radiation would contribute to proper radioprotection and reduce health hazards. Limitations: The study used physicians. Technologists perform most of the patient work in nuclear medicine and conventional radiology and it would have been more accurate to include technologists in the sample group. The control sample was only 26% of the total.
(Sari-Minodier et al. 2007)	France	Micronucleus *Radiology workers* 14.90 ± 8.10 *Controls* 11.80 ± 6.50 (*p =* .011)	Main conclusion: Besides ionizing radiation, modern hospitals have several chemical pollutants that may have genotoxic effects making it difficult for accurate cytogenetic monitoring of occupationally exposed technologists. Technologists must be educated and made aware of the importance of good radiation protection practices. Limitations: 57.6% of the exposed group and 46.4% of the control group had one or more medical irradiation as patients and the study estimated their exposure. It may be better to try and recruit people that have had no recent medical irradiation.
(Engin et al. 2005)	Turkey	Sister chromatid exchange per cell *Nuclear medicine* 11.4 ± 0.68 *Control* 4.17 ± 0.32 (*p* < .05) Apoptosis % *Nuclear medicine* 3.65 ± 0.25 *Control* 1.14 ± 0.08 (*p* < .05)	Main conclusion: The SCE test may be a valuable test to look for genotoxic effects of radiation exposure. Chronic low dose ionizing radiation exposure may lead to formation of free radicals and oxidative stress that may cause DNA damage and mutagenicity. Radiation protection standards need to be observed and maintained by technologists. Limitations: The control sample was only 29% of the total. Sister chromatid exchange is not specific to radiation and can be as a result of various other causes like environmental exposures.
(Lalic 2005)	Croatia	Chromosomal aberrations Acentric fragments *Nuclear medicine* 1.24 × 10^-3^ *Control* 0.07 × 10^-3^ *p <* .001 Dicentrics *Nuclear medicine* 0.12 × 10^-3^ *Control* 0	Main conclusion: Technologists frequently enter the cobalt bomb room, to position patients. At that time radiation is switched off but the air is ionized, and the patient’s body also emits radiation. There was higher aberration frequency in persons exposed to gamma rays. Regulatory measures may include shortening working hours and improving protection from radiation, besides the existing safety at work measures. Limitations: Gamma ray exposure only mentions ^60^Co and ^137^Cs. This would imply a radiotherapy unit and radiotherapists aren’t exposed to the wide ranges of radiation nuclear medicine technologists get because patients are the sources of most of the radiation in nuclear medicine. Control group may have been exposed prior because they were coming in for pre-employment checks before being employed in ionizing radiation zone.
(Thierens et al. 2000)	Belgium	Micronucleus with fluorescence in situ hybridization *Radiology workers* 21.88 ± 13.46 *Control* 18.63 ± 7.53 (*p =* .10) Micronucleus centromere positive *Radiology workers* 14.74 ± 11.71 *Controls* 11.22 ± 6.98 (*p =* .04) Micronucleus centromere negative (radiation induced) *Radiology workers* 7.15 ± 4.01 *Controls* 7.41 ± 3.48 (*p = .7*)	Main conclusion: Radiation induced micronuclei are mostly centromere negative suggesting a clastogenic action of radiation. The elevated number of centromere positive micronuclei points to a possibility of aneugenic effects after long term chronic exposure. Limitations: The control group has dose readings which implies some radiation exposure.

**Figure 1.**
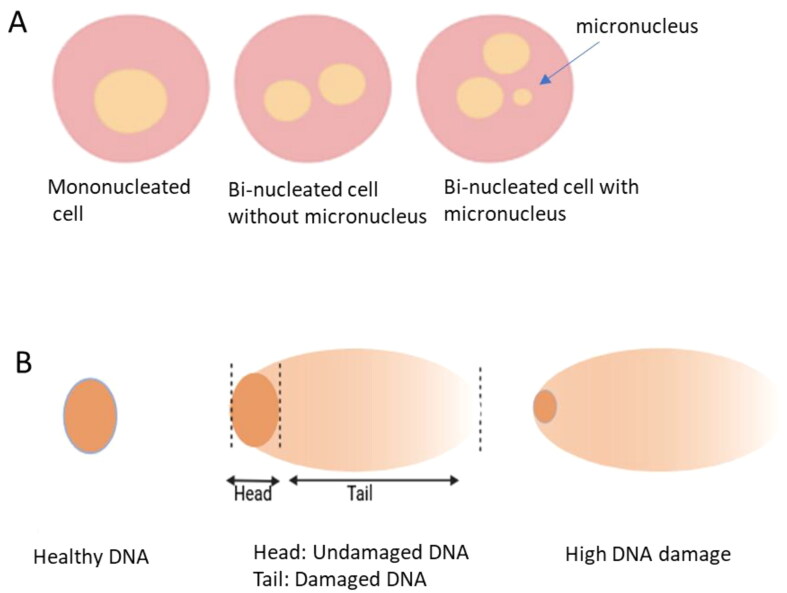
Analysis of DNA damage in lymphocytes using the micronucleus assay (A) and comet assay (B). Created with BioRender.com.

Most of these studies use the micronucleus assay (Thierens et al. 2000; Joseph et al. 2004; Sari-Minodier et al. 2007; Sahin et al. 2009; Zakeri and Hirobe 2010; Pajic et al. 2011; Ropolo et al. 2012; Bouraoui et al. 2013; Terzic et al. 2015; Vral et al. 2016; Gharibdousty et al. 2017; Miszczyk et al. 2019; Gao et al. 2020), all of which showed a significant increase in micronucleus frequency in technologists compared to the control groups. The micronucleus assay (Figure 1A) is quick but not very sensitive at doses below 0.3 Gy with a high frequency of background spontaneous micronuclei in the general population (Thierens et al. 1991). To increase sensitivity, Vral et al. (Vral et al. 2016) and Thierens et al. (Thierens et al. 2000) combined the micronucleus assay with fluorescent in situ hybridization to discriminate between radiation-induced centromere-negative micronuclei and spontaneous centromere positive micronuclei which mostly contain lagging chromosomes. The studies did not show a significant difference in the frequency of radiation-induced centromere-negative micronuclei in exposed versus unexposed volunteers.

DNA damage was also analyzed using the comet assay (Kopjar and Garaj-Vrhovac 2005; Georgieva et al. 2008; Martinez et al. 2010; El-Fayoumi et al. 2012; Dobrzyńska et al. 2014; Gaetani et al. 2018). The comet assay investigates the formation of single and double strand breaks, which can be caused by factors beyond radiation only (Figure 1B). There was a significant difference between exposed workers and controls in the comet score and tail length. An analysis (Martinez et al. 2010) of radiotherapy, nuclear medicine and general radiology technologists showed more DNA damage in nuclear medicine and radiotherapy technologists compared to general radiology. Interestingly, looking at spontaneous and induced DNA repair after in vitro irradiation of lymphocytes at 2 Gy (Georgieva et al. 2008) showed that levels of radiation below twice the dose from natural background increases radio resistance by priming the repair system (hormesis). A different study (Sahin et al. 2009; Dobrzyńska et al. 2014) also showed an increase in DNA damage in the leukocytes of nuclear medicine technologists, with more damage in those working in PET.

**Figure 2.**
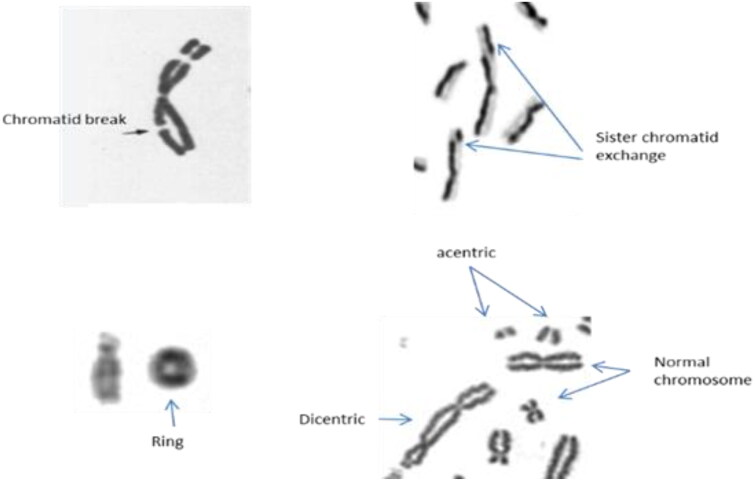
Types of chromosomal aberrations, e.g. chromatid break, ring, sister chromatid exchanges and dicentric and acentrics.

In terms of chromosomal damage, the frequency of sister chromatid exchange (SCE, Figure 2) has often been analyzed and shown to be higher in technologists than in controls (Bozkurt et al. 2003; Engin et al. 2005; Sahin et al. 2009). Equally, comparing SCE and micronuclei formation in nuclear medicine technologists before and after a one month vacation showed sufficient reduction in damage after the vacation, perhaps by DNA repair or by removal of damaged cells from the blood through apoptosis (Sahin et al. 2009). Damage was thus reversible after a vacation with no exposure, but it did point to the need for better radiation safety measures, as well as the short-term nature of DNA damage biomarkers and inadequacies around using SCEs or micronuclei as biomarkers of long-term damage. Also, SCEs and micronuclei assays are not specific to radiation. For example, various physical and chemical agents, and some chronic illnesses, increase the frequency of SCE (Sahin et al. 2009).

Several studies have also carried out using chromosomal aberration assays, which look at chromatid breaks, acentric fragments and dicentrics (Figure 2); these all showed a higher level of aberrations in technologists than the control group (Mozdarani et al. 2002; Lalic 2005; Zakeri and Hirobe 2010; El-Fayoumi et al. 2012; Pinto and Amaral 2014; Djokovic-Davidovic et al. 2016). These, including ring aberrations, are amongst the most robust methodology used in radiation research studies. In lymphocytes, these are predictive of the risk of cancer as seen in a multicenter study of 22,358 subjects across eleven countries (Bonassi et al. 2008). Another large-scale study in over 2,000 participants occupationally exposed to <20 mSv/yr of ionizing radiation, found that the presence of acentric fragments and dicentric chromosomes in lymphocytes correlated with a significant increase in cancer risk (Fucic et al. 2016).

### 3.2. Additional markers of radiation exposure

The above studies show that although physical dosimetry is routinely used to monitor exposure levels, determining actual biological effects of an individual may contribute to more accurate and comprehensive occupational risk assessments (Miszczyk et al. 2019). Biomonitoring of occupationally exposed technologists is a sensitive way to asses possible genotoxic effects of radiation exposure (Bouraoui et al. 2013) and can be used to detect early damage. Ideal biomarkers should be easy to collect and analyze, easily correlated to dose readings, and not affected by confounders (Pandey et al. 2010). Potential biomarkers that would be employed include γH2AX as an early marker for DNA damage and dicentric chromosomes as a marker of relatively long-term and stable chromosomal damage.

**Figure 3.**
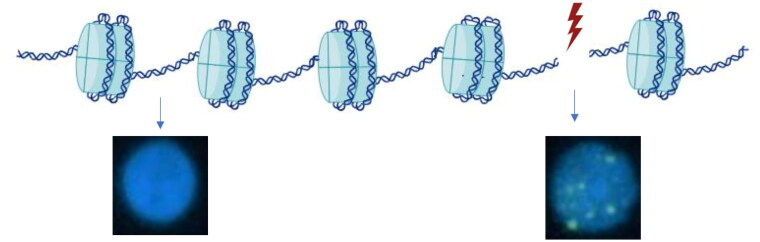
Injury to the cell, e.g. through radiation exposure causes double strand breaks (DSBs), in the DNA wrapped around the histones. This phosphorylates the histone and γH2AX can be visualized through immunofluorescence labeling. The cell on the left has no damage. The cell on the right has damage, the DNA is stained blue by DAPI and the γH2AX stained green marks DSB post irradiation to the cell. Created with BioRender.com.

The γH2AX assay provides important information regarding DNA damage post ionizing radiation exposure and its utility in patient populations has now also been demonstrated (El-Sayed et al. 2017; Hall et al. 2017). DNA is condensed into chromatin, which is wrapped around a set of eight proteins called histones (Ren et al. 2022). The histones have a positive charge which allows the negatively charged DNA to wind tightly around the histones. The H2A histone has a variant called H2AX, which is phosphorylated generating γH2AX when double strand breaks (DSBs) are created through, for example, radiation exposure (Figure 3). These DSBs need to be repaired and one early step in the repair of DSBs induced by ionizing radiation is the accumulation of phosphorylated proteins like the γH2AX at the site of damage (Hall et al. 2017). Phosphorylation gives the histone a negative charge which gives the DNA a more open formation so it can be repaired. γH2AX can be detected and quantified with the levels of γH2AX in the cell peaking half an hour after radiation exposure (El-Sayed et al. 2017; Hall et al. 2017) gradually decreasing to normal levels within 24-48 hours as the cells repair the damage (Młynarczyk et al. 2022) and able to detect DNA damage at radiation doses as low as 1 mGy (Khan et al. 2018). Limitations of the γH2AX include variability between different people in the induction of DSBs, variability in speed of repair leading to variations in γH2AX foci over time and confounding factors such as age, sex, ethnicity and lifestyle which have been shown to affect the number of γH2AX foci (El-Sayed et al. 2017; Hall et al. 2017; Młynarczyk et al. 2022).

The formation of dicentrics is mostly linked to exposure to radiation and only a few drugs that imitate the effects of radiation induce them (Lee et al. 2019). Misrepair of DSBs causes chromosome rearrangement and unstable aberrations like dicentric chromosomes, which have two centromeres (Figure 2). Most dicentrics are formed quickly within 2 hours post irradiation but due to their unstable nature they will reduce in time after exposure with a half-life of approximately 3 years (Moquet et al. 2018). Chromosomal aberrations, especially dicentrics and acentric fragments are the best identifiers of the effects of low dose ionizing radiation exposure in health care workers (Baudin et al. 2021). Dicentric chromosome assays (DCA) are the gold standard for radiation exposure monitoring with a sensitivity in the order of 0.1 Gy when analyzing up to 1000 cells (Hall et al. 2017; Moquet et al. 2018) and can be useful for predicting the risk of future radiation-induced health issues (Bonassi et al. 2008; Fucic et al. 2016). Analysis of dicentric chromosomes requires a large amount of experience and the assay takes time to run and to score a large number of cells (Hall et al. 2017) but as a long-lasting measure of radiation exposure, dicentrics are the most valuable biomarker of radiation dose exposure.

## 4. Future scope for biomarkers in radiation protection

Biomarkers of radiation exposure and effect have been suggested to be of potential use in studies of long-term risks associated with low dose radiation exposure. Molecular epidemiological studies have the potential to improve research in molecular biomarkers to improve our knowledge of ionizing radiation effects such as cataracts, cancer and cardiovascular effects (Hall et al. 2017; Ainsbury et al. 2023). The epidemiological studies reviewed show that health effects are observed after a long period of follow up by which time detrimental effects may already have occurred, as evidenced by the reduction in dose limits to the eyes due to cataract formation. The biomarker studies reviewed show the significance of biomarkers that provide information on the actual biological risk of radiation exposed workers as such data will not be obtained from physical dosimetry (Kopjar and Garaj-Vrhovac 2005) with an important advantage of biomarkers being that the individual radiation damage is measured including the variability of individual radiosensitivity. The health risks of chronic low dose ionizing radiation exposure are not totally understood and is further confounded by other factors like the doses of tracers administered, time in employment, type, and energy of radiation exposure. Preliminary and periodic biomonitoring exams, in this case the micronucleus assays, are already compulsory in Serbia (Djokovic-Davidovic et al. 2016). Such monitoring in other countries could possibly be utilized alongside physical dosimetry. More sensitive biomarkers like the γH2AX assay would need to be validated and simplified for routine biomonitoring first, whereas the dicentric assay is ready-to-go and would only need to be performed every two years due to its half-life in circulating lymphocytes.

Physical dosimeter readings depend on the placement of the dosimeter and measure the dose to the dosimeter material and not to the individual, the readings are calculated to convert the measured physical dose to the organ or whole-body dose. Biodosimetry on the other hand will detect changes at the cellular level that may give an indication of future health risks. Several of the studies reviewed highlighted the importance of biological indicators of radiation exposure in occupational settings as such data is lacking from physical dosimetry measurements and may personalize radiation risk (Mozdarani et al. 2002; Kopjar and Garaj-Vrhovac 2005; El-Fayoumi et al. 2012; Pinto and Amaral 2014; Djokovic-Davidovic et al. 2016; Vral et al. 2016; Gharibdousty et al. 2017).

There is a further potential use of biomarkers in nuclear medicine patients undergoing molecular radiotherapy (MRT) also known at targeted radionuclide therapy. MRT delivers radiation to targeted cancer cells by attaching a radioactive isotope to a ligand specific to the kind of cancer cells. The conventional protocol for treatment involves an initial baseline scan and then administering a standard dose sometimes adjusted for weight to all patients. The patient is then scanned after treatment to determine level of uptake and retention of radioligand. A shortfall of this protocol is the need to deliver the highest dose to the tumor while avoiding damage to normal cells. To overcome this, image based dosimetry has been suggested as a way to minimize damage to healthy tissue (Gear et al. 2020) by scanning the patient and quantifying the absorbed dose based on the distribution of activity within the scan and adjusting the dose administered for subsequent treatments if necessary. Biodosimetry could complement image based dosimetry by considering the radiosensitivity of the patient which varies between individuals and therefore help in determining the minimum dose to be administered for each patient while still achieving the best treatment outcome (Bolcaen et al. 2023). Bolcaen et al. identify studies that have used the DCA, micronucleus and the γH2AX assay to assess therapy effects. The DCA was limited to ^131^I-based therapies but allowed for cumulative dose estimations from prior treatments with the possibility of performing retrospective dosimetry up to two years after therapy. The micronucleus assay was also limited to ^131^I-based therapies but authors noted the wide variation in micronuclei counts making the assay more suitable for high therapy doses. The γH2AX assay was used in a wider variety of therapies including ^223^Ra and [^177^Lu]Lu-based therapies and could be used as a biomarker of cytotoxicity and also assess early response to treatment. Authors pointed out that it may be worthwhile to include long-term follow-up studies to investigate whether high residual γH2AX values are associated with acute bone marrow suppression and secondary blood malignancy. Lymphocytes can be considered as ‘circulating dosimeters’ because they are long living cells circulating across the whole body and they retain the signs of any radiation damage for longer periods of time (Bolcaen et al. 2023) making them a suitable addition for nuclear medicine patients undergoing therapy. It is our vision that dose-effect relationships can and should be personalized for nuclear medicine technologists using any or all of the biomarkers of radiation described here.

## 5. Conclusion

There is a clear detrimental link between high doses of radiation and health risks. Regulations on radiation exposure limits are constantly changing and based on the ICRP work, and as applied in the current system of radiation protection, there is a clear justification for protection against doses likely to cause tissue reactions and for limiting the exposures to lower doses as far as reasonably achievable (ICRP 118, 2012). However, despite a renewed focus on research in this area in recent years, the link between chronic low-dose exposure in nuclear medicine technologists and health risk using radiation-related biomarkers as a proxy remains relatively unexplored. There is a gap in the research on the relationship between radiation exposure and biological effects, specifically at low occupational doses and for imaging and nuclear medicine technologists. Expanding existing dosimetry and exposure assessment to include biomarkers of exposure to analyze DNA or chromosomal damage may allow for future regulations to follow radiation exposure (and effects) that are personalized for the technologist being monitored. This review of the available studies point to the need for further work to identify and characterize biomarkers in individuals exposed to ionizing radiation in nuclear medicine settings.
